# Antimicrobial Susceptibility Testing of *Leptospira* spp. in the Lao People’s Democratic Republic Using Disk Diffusion

**DOI:** 10.4269/ajtmh.18-0955

**Published:** 2019-03-18

**Authors:** Jennifer Boss, David A. B. Dance, Anisone Chanthongthip, Paul N. Newton, Vanaporn Wuthiekanun, Matthew T. Robinson

**Affiliations:** 1 Lao-Oxford-Mahosot Hospital-Wellcome Trust Research Unit (LOMWRU), Microbiology Laboratory, Mahosot Hospital, Vientiane, Lao People’s Democratic Republic;; 2 Faculty of Infectious and Tropical Diseases, London School of Hygiene and Tropical Medicine, London, United Kingdom;; 3 Nuffield Department of Medicine, Centre for Tropical Medicine and Global Health, University of Oxford, Oxford, United Kingdom;; 4 Mahidol-Oxford Tropical Medicine Research Unit, Faculty of Tropical Medicine, Mahidol University, Bangkok, Thailand

## Abstract

Leptospirosis is a global zoonotic disease caused by pathogenic bacteria of the *Leptospira* genus, which are fastidious, slow-growing organisms. Antimicrobial susceptibility data are limited; traditionally, the organisms have not been culturable on solid media. The recent development of *Leptospira* Vanaporn Wuthiekanun (LVW) agar, which facilitates rapid growth of *Leptospira* spp., provides the opportunity for antimicrobial susceptibility testing. Eighty-three *Leptospira* spp. clinical isolates originating from patients in Laos between 2006 and 2016 were tested against six antimicrobials (azithromycin, ceftriaxone, ciprofloxacin, doxycycline, gentamicin, and penicillin G) using disk diffusion on LVW agar. Quality control was undertaken using American Type Culture Collection (ATCC) reference strains with known susceptibilities on both standard media and LVW agar. All *Leptospira* spp. isolates produced large zones of inhibition around each of the six antimicrobials. All zones were greater than 25 mm: gentamicin produced the smallest zones (median 35 mm; interquartile range 30 mm–37 mm) and azithromycin produced the largest zones (median 85 mm; interquartile range 85 mm–85 mm). Zones produced by non-leptospiral ATCC reference strains on LVW agar were within 2 mm of accepted strain-specific quality control range on standard media. Antimicrobial activity on LVW agar appears to be similar to that on standard media. As there are no published susceptibility guidelines for the *Leptospira* genus, zone interpretation was subjective. *Leptospira* Vanaporn Wuthiekanun agar enabled antimicrobial susceptibility testing of multiple *Leptospira* isolates on solid media; the large zone sizes observed suggest that resistance has not emerged to these six antimicrobials in Lao *Leptospira* spp.

## INTRODUCTION

Leptospirosis is a widespread zoonosis caused by spirochete bacteria of the genus *Leptospira*. Although present worldwide, the highest disease burden is in tropical and subtropical areas, including Southeast Asian countries such as the Lao People’s Democratic Republic (Laos), where it is commonly under-recognized.^1^ The infection is spread to humans through environmental contamination by the urine of infected animals and causes a spectrum of clinical presentations ranging from mild febrile illness to severe disease that can result in organ failure and death.^2^ First-line therapy for severe leptospirosis is intravenous penicillin with oral doxycycline or azithromycin indicated in less-severe disease.^3^ Because of limited diagnostic capability and nonspecific clinical presentation, leptospirosis patients are commonly treated empirically for undifferentiated fever usually with beta-lactams, doxycycline, gentamicin, or ciprofloxacin.^4–8^

Antimicrobial susceptibility testing is used to determine the in vitro activity of agents against microorganisms and several different methods are available.^9^ Agar-based culture methods are a mainstay in susceptibility testing for their ability to facilitate growth of many pathogens within 24 hours.^10^ However, susceptibility testing of *Leptospira* spp. has been limited as leptospires are fastidious, slow-growing organisms unable to grow on standard laboratory media.^10,11^ There has thus been no robust method of culturing leptospires on solid media and no standard method for in vitro testing of antimicrobial agents against *Leptospira* spp.^10–12^ Culture using special liquid or semiliquid media can be performed but is labor intensive, requiring expertise and may take weeks to months to achieve growth.^10,13^ Both macro- and micro-dilution methods have been used to test the susceptibility of isolates from humans and animals, but they are time consuming and difficult to control.^10,11^ Both clinical evidence and in vitro studies suggest that leptospires are susceptible to beta-lactams, macrolides, tetracyclines, and fluoroquinolones, but the lack of large-scale studies means there is little information about the prevalence of antimicrobial resistance within the genus.^14–23^

The first solid medium for *Leptospira* spp. named *Leptospira* Vanaporn Wuthiekanun (LVW) agar, which facilitates rapid growth, was developed in 2013.^10^ The culture of *Leptospira* spp. using LVW agar entails initial incubation at 30°C in 5% CO_2_ for 2 days followed by incubation in ambient air at 30°C for 5 days, with growth visible by this time.^10^ A small pilot study demonstrated the use of LVW agar for antimicrobial susceptibility testing in disk diffusion assays.^24^

The aim of our study was to expand on this initial study and use disk diffusion assays on LVW agar to assess the antimicrobial susceptibility of 83 Lao human *Leptospira* spp. isolates to six antimicrobials (azithromycin, ceftriaxone, ciprofloxacin, doxycycline, gentamicin, and penicillin G), which are commonly used to empirically treat fever in Laos.^25–27^ To validate the use of disc diffusion susceptibility testing for leptospires on LVW agar, we compared the activity of the antimicrobials against control strains from the American Type Culture Collection (ATCC) on LVW agar to that on Mueller–Hinton agar.

## MATERIALS AND METHODS

### Clinical isolates.

Eighty-three *Leptospira* spp. isolates were tested from a collection of clinical isolates originating in Laos between 2006 and 2016. The isolates were isolated from blood clots of *Leptospira*-positive patients at four Lao hospitals (Mahosot Hospital and Friendship Hospital in Vientiane, Luang Namtha Provincial Hospital, and Salavan Provincial Hospital) during studies of the etiology of fever.^8^ The isolates were maintained in Ellinghausen–McCullough–Johnson–Harris (EMJH) broth at room temperature from the day of isolation and subcultured approximately every 6 months before use in this study. To maximize viability at the time of susceptibility testing, the isolates were subcultured before inoculation on LVW agar.

### Preparation of LVW agar and EMJH broth.

*Leptospira* Vanaporn Wuthiekanun agar was prepared as described by Wuthiekanun et al.^10^ Briefly, for 1 L of LVW agar, 2.3 g of *Leptospira* Medium Base EMJH (BD Difco, Sparks, MD), 10 g of Noble Agar (BD Difco), and 1 mL of 1% sodium pyruvate (Merck, Billerica, MA) solution were added to 800 mL of distilled water. After autoclaving, 100 mL of *Leptospira* Enrichment EMJH (BD Difco) and 100 mL of normal rabbit serum (Gibco, Brigg, United Kingdom) were aseptically added. The agar was poured, dispensing 25 mL into a 90-mm diameter Petri plate for a depth of 4 mm. Each batch of LVW agar was tested with an in-house clinical isolate of *Leptospira* spp. (UI130) to confirm that it supported growth of *Leptospira* spp. Ellinghausen–McCullough–Johnson–Harris broth was prepared following LVW agar preparation, with final concentration of agar at 0.1% and rabbit serum at 3%.

### Inoculation of ATCC reference strains on LVW agar and standard media.

The following strains were used to compare zone diameters produced on LVW agar with those on Clinical and Laboratory Standards Institute (CLSI) standard media: *Escherichia coli* ATCC 25922, *Staphylococcus aureus* ATCC 25923, *Pseudomonas aeruginosa* ATCC 27853, and *Streptococcus pneumoniae* ATCC 49619. All other conditions of disk diffusion were as defined by CLSI, except that Mueller–Hinton with 5% goat blood was used for *S. pneumoniae* ATCC 49619 instead of sheep blood which is not available in Laos.^28^ The zones produced on standard media and LVW agar were compared with the strain-specific quality control zone ranges on standard media.^28^ Note that only antimicrobials with published ranges for a specific strain were used.

### Estimation of culture concentration using microscopy.

Growth was monitored by dark-field microscopy (Olympus BX51, Tokyo, Japan) and assessed using a 0–4-point growth scale developed by Mahidol Oxford Tropical Medicine Research Unit (Bangkok, Thailand) to estimate *Leptospira* spp. viability and concentration in EMJH broth. Peak viability was denoted as 4+ growth and was visualized by high motility and dense coverage of leptospires across the field when viewing a 5-µL drop of culture with the ×10 objective. This indicated a leptospire concentration of approximately 1 × 10^8^ CFU/mL, the desired concentration for inoculation of LVW agar. The isolates were inoculated within 24 hours of reaching 4+ growth, after which time the culture ceased to be considered fresh as indicated by clumping of the bacteria.

### Inoculation, incubation, and application of antimicrobial disks.

Isolates that had attained 4+ growth were inoculated onto LVW agar by dispensing 300 µL of culture onto the center of a single plate and spreading the bacteria with a cotton swab using a rotary plater (Leetech, Bangkok, Thailand). Seven plates were inoculated with each isolate—one for each of the six antimicrobials (only one disk was applied to each plate as the zones were anticipated to be very large) and one growth control plate with no disk.

Once dry, the inoculated plates were inverted, placed in a candle jar (estimated 2–3% CO_2_),^29^ and incubated at 30°C for 48 hours.

Six antimicrobials (Table 1) were tested by disk diffusion as previously described.^24^ On day 3 of incubation, each disk (Oxoid^™^, Basingstoke, United Kingdom) was aseptically applied to the center of a single plate, apart from the control. The plates were inverted and incubated at 30°C in ambient air for 5–12 days, depending on individual growth rates.

**Table 1:** Six antimicrobials used for disk diffusion susceptibility testing

Antimicrobial	Disk content	Catalog number (Oxoid)
Azithromycin	15 µg	CT0906B
Ceftriaxone	30 µg	CT0417B
Ciprofloxacin	5 µg	CT0425B
Doxycycline	30 µg	CT0018B
Gentamicin	10 µg	CT0024B
Penicillin G	10 units	CT0043B

### Measuring growth and repeat inoculation of four isolates.

Growth was monitored from day 6 of incubation, and final measurements were taken between days 8 and 13, depending on individual growth rates. The zones of inhibition were measured from the innermost point of growth as seen with the naked eye. Any visible contamination or difficulty measuring zones was noted. Four isolates underwent repeat testing to assess reproducibility. The isolates were re-subcultured before the repeat inoculation.

## RESULTS

### Comparison of zone diameters on LVW agar and standard media.

All zones produced by *E. coli* ATCC 25922, *S. pneumoniae* ATCC 49619, *S. aureus* ATCC 25923, and *P. aeruginosa* ATCC 27853 on CLSI standard media were within the strain-specific quality control ranges published in CLSI guidelines (Table 2).^26^ The zones produced by these four reference strains on LVW agar were also within the strain-specific ranges for standard media except in the following cases. *Escherichia coli* produced zones 2 mm smaller than the minimum range value for ceftriaxone and doxycycline. *Streptococcus pneumoniae* produced a zone 1 mm larger than the maximum range value for azithromycin and 1 mm smaller than the minimum range value for doxycycline. Finally, *S. aureus* produced a zone 2 mm larger than the maximum range value for azithromycin.

**Table 2:** Zones of inhibition (mm) of ATCC reference strains on CLSI standard media and LVW agar^28^

Antimicrobial	*Escherichia coli* ATCC 25922			*Staphylococcus aureus* ATCC 25923			*Pseudomonas aeruginosa* ATCC 27853			*Streptococcus pneumoniae* ATCC 49619		
	CLSI range	Mueller–Hinton	LVW	CLSI range	Mueller–Hinton	LVW	CLSI range	Mueller–Hinton	LVW	CLSI range	Mueller–Hinton with 5% goat blood	LVW
Azithromycin	–	–	–	21–26	26	28	–	–	–	19–25	25	26
Ceftriaxone	29–35	34	27	–	–	–	–	–	–	30–35	33	31
Ciprofloxacin	–	–	–	–	–	–	25–33	30	30	–	–	–
Doxycycline	18–24	24	16	–	–	–	–	–	–	25–34	31	24
Gentamicin	19–27	23	24	19–27	26	25	17–23	21	23	–	–	–
Penicillin G	–	–	–	26–37	37	26	–	–	–	24–30	26	24

ATCC = American Type Culture Collection; CLSI = Clinical and Laboratory Standards Institute; LVW = *Leptospira* Vanaporn Wuthiekanun. Note that only antimicrobials with published ranges for a specific strain were used. Clinical and Laboratory Standards Institute quality control ranges on standard media are also given.

### Growth on LVW agar.

The *Leptospira* spp. isolates grew at variable rates on LVW agar with growth appearing between day 8 and 13 of incubation. Zones of inhibition were often not distinct until 1 or 2 days after growth initially appeared. Approximately half of the inoculated plates could be measured on day 8.

**Figure 1.**
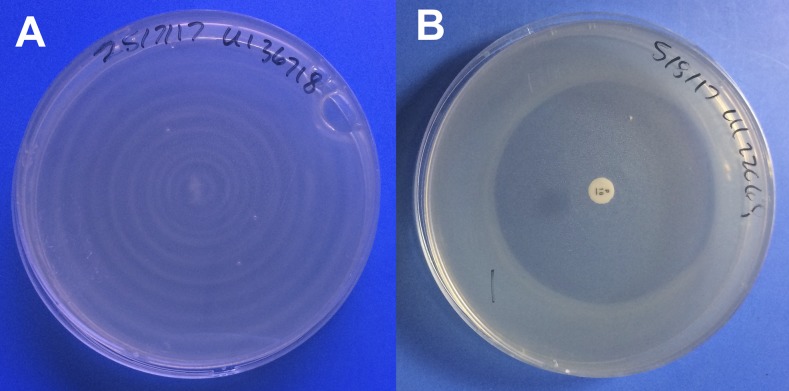
Growth of *Leptospira* spp. isolates on *Leptospira* Vanaporn Wuthiekanun agar. (**A**) Growth control of isolate UI36718 with no antimicrobial disk. (**B**) Zone of inhibition (55 mm) of isolate UI22068 around penicillin G. This figure appears in color at www.ajtmh.org.

Once visible, there was variability in the appearance of the growth. Although all growth was subsurface and had a white cloud-like appearance (Figure 1), there was a range in the distinctness and apparent density of growth. In particular, 18 isolates had fainter growth across all plates, although zones of inhibition were still distinguishable. All isolates had visible growth on the growth control plate except in three cases where there was either widespread surface plate contamination (two isolates) or insufficient culture volume to inoculate the control (one isolate).

### Large zones of inhibition produced by *Leptospira spp*. isolates.

**Figure 2.**
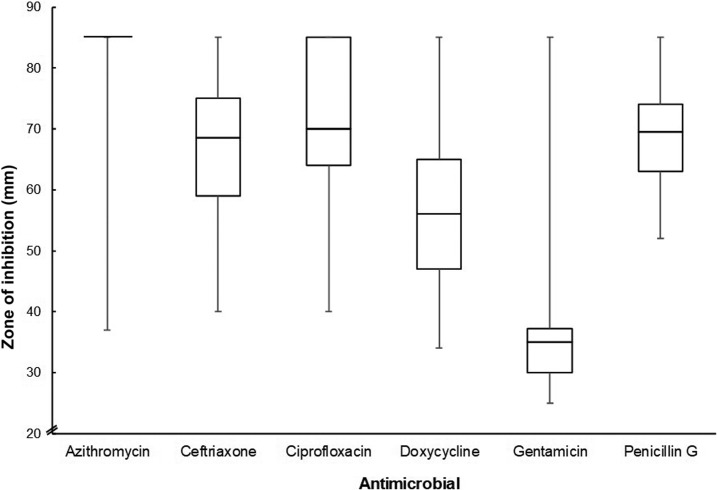
Median zones of inhibition (mm) produced by *Leptospira* spp. isolates to six antimicrobials on *Leptospira* Vanaporn Wuthiekanun agar. The box indicates interquartile range with median and bars indicate minimum and maximum values. The interquartile range, median, and maximum zone of azithromycin lie on the same value.

All *Leptospira* spp. isolates produced zones more than 25 mm in diameter for each of the six antimicrobials (Figure 2). Plates on which no growth was visible (but with growth on the corresponding control) were assumed to have a zone of 85 mm or larger, the diameter of the plate base. Gentamicin produced the smallest zones, with a median zone diameter of 35 mm (interquartile range 30 mm–37 mm) and a range between 25 and 85 mm. Azithromycin produced the largest zones with a median zone diameter of 85 mm (interquartile range 85 mm–85 mm) and a range between 37 and 85 mm (note that the interquartile range, median, and maximum zone of azithromycin lie on the same value). Ceftriaxone, ciprofloxacin, doxycycline, and penicillin G produced intermediate zone medians at 69, 70, 56, and 70 mm, respectively. No plate had growth within 25 mm of any disk.

#### Zone interpretation.

**Figure 3.**
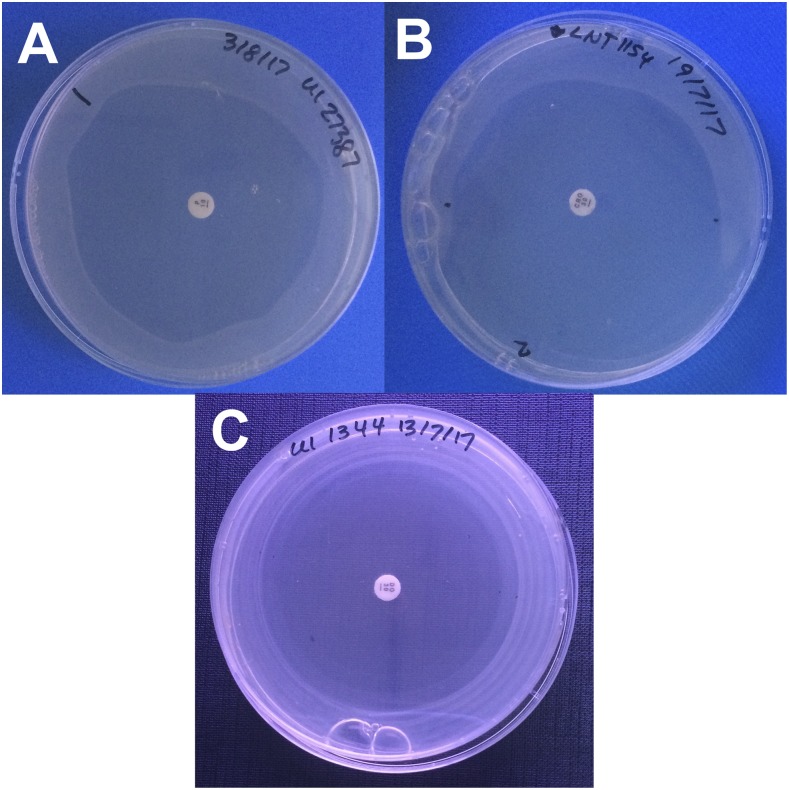
Difficult to measure zones of inhibition of *Leptospira* spp. isolates on *Leptospira* Vanaporn Wuthiekanun agar. (**A**) Zone that is not perfectly round. (**B**) Zone that does not extend to a full circle. (**C**) Multiple zones. This figure appears in color at www.ajtmh.org.

Eight percent of zones were noted as difficult to measure (Figure 3). These included zones that were not perfectly circular (for which a mean of two measurements was calculated) and zones which did not extend to a full circle (for which the radius was measured and doubled if a diameter could not be read). Also included were zones which were difficult to distinguish from growth resulting from excess fluid build-up at the edge of the plate during inoculation. This occurred most often with very large zones almost reaching the plate edge. In addition, azithromycin produced zones with very faint outlines, even if the rest of the plates for a given isolate had distinct growth and zones.

### Repeat testing of four isolates.

Four isolates were tested multiple times to assess reproducibility of the method. Isolate LNT3110 was tested twice, and zones for each antimicrobial between replicates were within 3 mm of each other (Table 3). The zones produced by LNT2714 had differences of more than 5 mm between replicates for each antimicrobial. The zones produced by UI27257 had differences of more than 8 mm between replicates for each antimicrobial with a large difference of 34 mm between zones for ceftriaxone. Isolate FS3849 was tested three times and ranges between the maximum and minimum zone diameters produced in the three tests were 15, 18, 19, 7, 8, and 21 mm for azithromycin, ceftriaxone, ciprofloxacin, doxycycline, gentamicin, and penicillin G, respectively.

**Table 3:** Zones of inhibition (mm) produced by repeat disk diffusion testing of four *Leptospira* spp. isolates on *Leptospira* Vanaporn Wuthiekanun agar

Isolate code	Test	Growth control	Azithromycin	Ceftriaxone	Ciprofloxacin	Doxycycline	Gentamicin	Penicillin G	Time (days) to 4+ growth
LNT3110	1	+	85	75	60*	61†	38	77	4
	2	+†	85	75	Widespread contamination	63	35	75*	3
LNT2714	1	+	78*	40	55	49†	28	60	4
	2	+	85	65	69	65	36	65	3
UI27257	1	+	85	50	61	45	30	53	3
	2	+	85	84	75	60	38	66	3
FS3849	1	+	85†	58	55	50	27	54	4
	2	+	70*	47	53*†	43	31	62†	9
	3	+†	85	65	72	50	35	75	3

Also included is the time to reach 4+ growth following subculturing. Growth on the control plate without an antimicrobial disk is indicated by a plus (+) sign.

* Zone difficult to measure.

† Surface contamination.

## DISCUSSION

This study, testing 83 clinical isolates of *Leptospira* spp. collected over a 10-year period against six antimicrobials, is the largest study to date we are aware of to use disk diffusion assays on LVW agar for susceptibility testing of *Leptospira* spp.^10,12,24,30^ The data suggest that all isolates were susceptible to the six antimicrobials and that the agents were highly active in LVW agar. There is, thus, no evidence that leptospires in Laos have developed resistance to standard antimicrobial therapy.^4–8^

All *Leptospira* spp. isolates in this study produced large zones of inhibition, indicating high sensitivity to the six antimicrobials. As there are no published susceptibility guidelines for *Leptospira* spp., interpretation of the zone sizes is subjective and the isolates cannot be definitively classed as susceptible, intermediate, or resistant.^10,11,31^ However, the large zone sizes suggest a high level of antimicrobial activity in comparison with the zone sizes that would be expected with other bacteria according to CLSI guidelines.^28^ It should be noted, however, that the longer incubation period needed to allow growth of *Leptospira* spp. is likely to have resulted in larger zone diameters than would be observed for faster growing organisms. The similar zone sizes produced by ATCC reference strains on both standard and LVW agar, where no zone was more than 2 mm outside the respective CLSI range, suggest that the antimicrobials have similar activity in both media.

There was a range of zone sizes produced by *Leptospira* spp. isolates for each antimicrobial. Although these ranges may represent slight differences between the susceptibilities of individual isolates to the antimicrobials, the zones were still very large, suggesting that all isolates were susceptible. Gentamicin, which generally diffuses poorly through media and, thus, tends to produce smaller zones than some other agents, produced the smallest zones.^28^ Azithromycin produced the largest zones, with many isolates demonstrating no visible growth, thus indicating a zone greater than the diameter of the plate. The four other antimicrobials used in this study had zone ranges intermediate between gentamicin and azithromycin, but all large relative to CLSI susceptibility breakpoints for other organisms.^28^ The four isolates were tested multiple times to assess reproducibility. The results suggest that although there is considerable inherent variability in the method, it does not detract from the key observation that no obvious resistance was detected among the isolates.

Several elements of the methodology contributed to the qualitative nature of the study. First, zone interpretation was subjective with growth often difficult to visualize on the agar. Cultures of *Leptospira* spp. are different in appearance from many other bacteria on solid media as growth is subsurface and has a hazy, cloud-like appearance; therefore, many plates had zones that were difficult to measure.

Second, determination of the inoculum concentration before inoculation was also subjective. Dark-field microscopy was used to assess isolate growth with “4+ growth” presumed to indicate a concentration of 1 × 10^8^ CFU/mL. This visual assessment is dependent on drop thickness on the microscope slide and made more difficult by varying leptospire lengths in different *Leptospira* isolates*.* Irregularity in inoculum concentration can affect zone size, for example, where higher concentrations can result in comparatively smaller zone sizes across all antimicrobials as exhibited by isolates FS3849 and SV588.

The *Leptospira* spp. isolates exhibited individual growth rates as demonstrated by variation in both the time to reach 4+ growth after subculturing and time for visible growth to appear on LVW agar. Although the median time to reach 4+ growth was 5 days, 21 isolates were fast growing taking 3 days and two isolates were slow growing taking 12 days to reach 4+ growth. In addition, 18 isolates exhibited discernibly fainter growth on LVW agar, even when measuring zones on day 13 of incubation. Given the possibility of inoculum concentration variability, it cannot be determined if the faint growth observed was due to low inoculum concentration or slow isolate growth rate.

Contamination, the source of which was unclear, was a problem throughout the study although zones could still usually be read. The long duration of incubation meant that contamination was more problematic than in the testing of more rapidly growing pathogens. Most isolates could only be tested once because of resource constraints.

Our data highlight that although antimicrobial susceptibility testing of *Leptospira* spp. is challenging and a suitable consensus methodology is required,^12^ the use of disk diffusion assays on LVW agar suggest that clinical *Leptospira* spp. isolates in Laos remain susceptible to standard antimicrobial therapy. These results are consistent with the limited body of evidence of *Leptospira* spp. susceptibility using broth macro- and micro-dilution, which also suggest a lack of leptospire resistance to commonly prescribed antimicrobials.^6,7,12,14–17,19–23,32,33^ This may be related to the fact that the normal reservoirs of leptospires are environmental sources and wildlife, which generally have minimal selective pressure from antimicrobials, unless they are associated with sewage outlets or watercourses where antimicrobial residues may be found.^33^ There are thus no implications from this study that empirical or definitive treatment strategies for leptospirosis in Laos need to be modified.

## Supplementary Files

Supplemental material
