# Supplementary material for: Antimicrobial Susceptibility Testing of Leptospira spp. in the Lao People’s Democratic Republic Using Disk Diffusion
Source: Am J Trop Med Hyg. 2019 Mar 18;100(5):1073–8. doi: 10.4269/ajtmh.18-0955 (PMC6493943; doi:10.4269/ajtmh.18-0955)
Supplement: Supplementary file 1 [file tpmd180955.SD1.pdf]

The following are supplemental materials and will be published online only

Supplementary materials

S1 Table. Zones of inhibition (mm) produced by *Leptospira* spp. isolates for 6 antimicrobials on LVW agar. Also included is the time to reach 4+ growth following subculturing and observation of faint growth. Time to reach 4+ growth is also given for 6 plates of different isolates where the isolate was re-subcultured and tested at a separate time. Basic statistical computations for each antimicrobial such as median and mode are located at the bottom of the table.

Isolates codes: UI = patient admitted at Mahosot Hospital, Vientiane; LNT = admitted at Luang Nam Tha Hospital; SV = admitted at Salavan Hospital; FS = admitted at Friendship Hospital, Vientiane.

+ Growth on the control without an antimicrobial disk;

* Surface plate contamination;

† Difficult to measure zones.

Isolate number	Isolate code	Growth control	Azithromycin	Ceftriaxone	Ciprofloxacin	Doxycycline	Gentamicin	Penicillin G	Time to reach 4+ growth (days)	Additional observations
1	UI8368	+	85	85	85	40	35	58	6	Faint growth
2	UI8414	+	85	59	70	85*	33	85	6	
3	UI8434	+	85	54	70*	51	30	65	5	Faint growth
4	UI8440	+	85	68	70	68	32	72	5	
5	UI8561	+*	85	40	85	85	85	75	6	
6	UI8596	+	85	75*	70	60	48	69	6	Faint growth
7	UI8704	+	85	60	70	55	36	60	6	
8	UI12344	+	85	64	57*†	34	30*	65*	6	
9	UI12342	+	85	85*	85	70	47	72	5	Faint growth

Isolate number	Isolate code	Growth control	Azithromycin	Ceftriaxone	Ciprofloxacin	Doxycycline	Gentamicin	Penicillin G	Time to reach 4+ growth (days)	Additional observations
10	UI12621	+	77	60*	65*†	59	26	63	12	
11	UI12764	+	85	70*	85*	70*	43*	80*	8	Faint growth
12	UI12758	+*	80	52	73	48	28	65	8	
13	UI12769	+	85	80	85	70	35	71	3	Faint growth
14	UI12830	+*	85	75	68	65	35	70	6	
15	UI13005	+	75	52	68	53	27	73†	12	
16	UI13016	+	85	60	65	50	35	68	3	
17	UI12823	Widespread Contamination	85*	65	85*	50	40	80	6	Faint growth
18	UI13087	+*	85	65†	45†	57*	30	60*	6	
19	UI13098	+	75	70†	55	45	43	70	5	Faint growth
20	UI13372	Widespread Contamination	75	64	65	60	34	69	8	
21	UI14346	+	85*	70*	n/a	n/a	33	70	5	
22	UI14469	+	85	75*	60	60	34	80	4	Faint growth
23	UI14535	+*	85	60	60	50	32	70	4	Faint growth
24	UI14631	+	85	76	70	70	35	80	8	
25	UI14721	+	85*	49*	64	45	31	70	9	
26	UI14851	+	85	80	70	74	37	76	8	Faint growth
27	UI15117	+	60	54	60*	50	40	70	4	
28	UI15191	+*	85	60	65	49	30	70	4	
29	UI15218	+	85*	70	Widespread Contamination	59	39	66	8	
30	LNT1154	+	85	64*†	67*†	60	31	59*	9	
31	SV387	+	37†	45	40*	45†	25	54	6	
32	UI15308	+	80	65	70	64	33	68	4	
33	UI15357	+	85	70	70	55	35	70	5	
34	UI17545	+*	85	70	79	51	39	64	4	

Isolate number	Isolate code	Growth control	Azithromycin	Ceftriaxone	Ciprofloxacin	Doxycycline	Gentamicin	Penicillin G	Time to reach 4+ growth (days)	Additional observations
35	UI17322	+	85*	74	85	67	32	73	3	
36	LNT1600	+*	85	59	65	45	32*	70	5	
37	LNT1612	+	85*	65	75 (4 days)	60	35 (4 days)	85	5	
38	LNT1671	n/a	85	75†	85	63†	36	85	4	Faint growth
39	UI18681	+	85*	70*†	69†	55	28*	74*†	9	
40	UI19245	+	85	70	85	85*	35	85	5	Faint growth
41	UI19893	+	85*	70	70	70†	30	74	3	
42	UI20184	+	68	63	69	59	35	68*	7	
43	LNT2340	+	85	71†	65	64	38	73†	6	
44	UI20403	+	85	72	85	60	35	72	3	
45	UI20802	+	85	85*	85	70	35	85	3	Faint growth
46	SV865	+*	85	60 (4 days)	70 (4 days)	47	35*	67	3	
47	UI11076	+*	85*	85*†	85*	69*	39	85*	6	
48	UI22068	+*	60	85*	64	51	25	55	3	
49	LNT2714	+	78†	40	55	49*	28	60	4	
	LNT2714	+	85	65	69	65	36	65	3	
50	UI22743	+*	85	58	85*	65	32*	65	3	
51	SV1038	+	85*	69	74*†	70*	36	75	4	
52	LNT2747	+	40†	56*	64	44	30	63	6	
53	UI23056	+	85*	56	66	50*	30	60	3	
54	UI23152	+*	85	70	80*	45	46	60	5	
55	UI23159	+*	85	85	73	62	36†	70	6	
56	LNT2825	+*	85	85	85	70	45†	80	4	
57	LNT2859	+	85	70*	85	42	30†	65	3	
58	UI25281	+*	75	55	56	45	30	52	3	
59	UI26021	+	85	82	78	75	42	71†	5	

Isolate number	Isolate code	Growth control	Azithromycin	Ceftriaxone	Ciprofloxacin	Doxycycline	Gentamicin	Penicillin G	Time to reach 4+ growth (days)	Additional observations
60	UI26702	+	85	85	85	65	42	85	3	Faint growth
61	UI27257	+	85	50	61	45	30	53	3	
	UI27257	+	85	84	75	60*	38	66	3	
62	UI27365	+	85	64	57	59	35	65	5	
63	UI27387	+	85	85	85*	65	39	67	3	Faint growth
64	LNT2987	+*	42†	62	60	46	33	75†	6	
65	UI27739	+*	85	56	70	56	31	55	5	
66	UI27845	+	85*	85	85	79	43	65	3	Faint growth
67	UI27877	+	85	70*	80	55	35	63	3	
68	FS3849	+	85*	58	55	50	27	54	4	
	FS3849	+	70†	47	53*†	43	31	62*	9	
	FS3849	+	85	65	72	50	35	75	3	
69	LNT3077	+	55	49	65	55	29	70	4	
70	LNT3086	+*	85*	60†	53†	48†	30	61	4	
71	LNT3102	+	75†	56	65†	35	31	54	6	
72	UI30454	+	85	60	85<br>(4 days)	45	35*	60	5	
73	LNT3110	+	85	75	60†	61*	38	77	4	
	LNT3110	+	85	75	Widespread Contamination	63	38	75†	3	
74	UI30711	+	85	85	65	40	35†	65	6	
75	UI30881	+	85	73	85	80	50*	85	3	
76	UI32865	+	74	40*	56	44*	27*	57	6	
77	UI33216	+*	85*	70*<br>(4 days)	63	40	32<br>(4 days)	68	5	
78	SV547	+	85	57*	54†	47†	28	58	6	
79	SV588	+	40	75†<br>(6 days)	55	37	32	56	6	
80	UI36718	+	75*	50	55	40*	29	57	6	

Isolate number	Isolate code	Growth control	Azithromycin	Ceftriaxone	Ciprofloxacin	Doxycycline	Gentamicin	Penicillin G	Time to reach 4+ growth (days)	Additional observations
81	UI36788	+*	85	70	75	60†	36 (5 days)	65†	4	
82	UI37443	+	85*	85	85	70	35	70	4	
83	UI37640	+	85	75	75	70	40	70	6	Faint growth
		**Median**	85	69	70	56	35	70	5	
		**IQR (1st to 3rd quartiles)**	85 to 85	59 to 75	64 to 85	47 to 65	30 to 37	63 to 85	4 to 8	
		**Mode**	85	70	85	70	35	70	6	
		**Minimum**	37	40	40	34	25	52	3	
		**Maximum**	85	85	85	85	85	85	12	
		**Range**	48	45	45	51	60	33	9	

IQR = interquartile range; LVW = *Leptospira* Vanaporn Wuthiekanun; n/a = not available.
